# GrafGen: distance-based inference of population ancestry for *Helicobacter pylori* genomes

**DOI:** 10.1186/s12859-025-06294-y

**Published:** 2025-11-26

**Authors:** William Wheeler, Difei Wang, Isaac Zhao, Filipa F. Vale, Yumi Jin, Charles S. Rabkin

**Affiliations:** 1 Information Management Services, Rockville, MD USA; 2 Division of Cancer Epidemiology & Genetics, National Cancer Institute, National Institutes of Health, Rockville, MD USA; 3 Axle Informatics, Rockville, MD USA; 4 BioISI—Instituto de Biossistemas e Ciências Integrativas, Faculdade de Ciências, Universidade de Lisboa, Lisbon, Portugal; 5 University of Pennsylvania, Philadelphia, PA USA; 6 Cancer Genomics Research Laboratory, Frederick National Laboratory for Cancer Research, National Institutes of Health, Rockville, MD USA

**Keywords:** *Helicobacter pylori*, Bacterial genomics, Population structure, SNP analysis; ancestry inference, Prophage diversity, Open-source software,Bioconductor, R package

## Abstract

**Background:**

*Helicobacter pylori* is a highly diverse gastric bacterium whose genomic variation both reflects human migration and complicates genome-wide association studies (GWAS). Its 1.67 Mb genome contains ~ 143,000 biallelic SNPs with minor allele frequency > 1%, making population stratification a major confounder. Existing model- and distance-based methods for bacterial ancestry classification often yield inconsistent results depending on dataset composition. A robust and generalizable framework is needed to improve downstream analyses.

**Results:**

We developed GrafGen, an open-source R package adapted from the human ancestry tool GrafPop, for the classification of *H. pylori* and prophage populations. Using reference data from the *H. pylori* Genome Project (1,011 genomes from 50 countries), GrafGen identified nine distinct bacterial populations and four prophage groups by genetic distance clustering. Validation with 255 GenBank sequences showed consistent mapping to GrafGen-defined populations. Classifications based on subsets of 14,300 and 1,430 SNPs achieved > 97% and > 90% concordance, respectively, with those using the full 143,000 SNPs, demonstrating robustness to down-sampling. The package integrates visualization tools for geometric interpretation of ancestry structure and is distributed via Bioconductor (v1.4.0, nine-population reference) and GitHub (v2.0_beta, general framework for haploid species and prophages).

**Conclusions:**

GrafGen provides a reliable approach for classifying *H. pylori* ancestry and correcting for bacterial population stratification in GWAS. By enabling more accurate inference of genotype–phenotype associations, the method enhances studies of bacterial genetics and host–pathogen interactions. The underlying algorithm is extensible to other haploid organisms with adequate reference data, broadening its relevance beyond *H. pylori*.

**Supplementary Information:**

The online version of this article (10.1186/s12859-025-06294-y) contains supplementary material, which is available to authorized users.

## Background

*Helicobacter pylori*, a bacterium widely associated with gastric cancer, is a genetically diverse organism with an average haploid genome of 1.67 Mb in a single circular chromosome [1]. The variable presence of the cytotoxin-associated antigen A (*cagA*) gene has been associated with increased gastric cancer risk [2]. Other genetic variations are potential additional risk factors warranting investigation. Likewise, prophages can significantly alter the pathogenicity and gene regulation of their bacterial hosts, shaping genome organization by promoting genome rearrangements, merging with other mobile elements, and accumulating pseudogenes. The phylogeographic structures of *H. pylori* and its symbiont prophage are organized into major populations, which correspond to patterns of ancient human migrations [3–5].

Confounding by population stratification is a major source of bias for genome-wide association studies (GWAS) [6]. Population stratification in bacterial genomes is particularly problematic for GWAS, as it can produce spurious associations that merely reflect geographic structure rather than true functional variants. For example, in prior studies of *H. pylori*, disease-linked SNPs were later shown to cluster with specific phylogeographic lineages, suggesting confounding rather than causation [7, 8]. Previous model- and distance-based adjustments for bacterial population structure yield differing results depending upon the strains included in analyses [9, 10]. Population assignment methods based on whole-genome sequencing (WGS) are fragmented and often challenging for the scientific community to use effectively.

Over the past decade, several tools have been developed to address bacterial population structure in a genomic context. These include the BAPS family of methods [11, 12] and PopPUNK [13], which enable rapid and scalable clustering of microbial genomes. However, these tools may require prespecification of cluster numbers, be sensitive to sample composition, or rely heavily on presence/absence matrices of accessory genes, which are highly variable and often incomplete in species such as *H. pylori*. Moreover, the recombinogenic nature of *H. pylori* [14, 15] can introduce challenges for alignment-based or model-driven methods. To address these limitations, we developed GrafGen—a distance-based, reference-anchored tool for ancestry classification. Adapted from the GrafPop tool used in human population genomics [6], GrafGen provides stable classification based on genome-wide SNP distances relative to fixed reference populations. This approach ensures reproducibility across datasets, even when genomes are incomplete or exhibit high levels of recombination. Moreover, GrafGen is also suitable for classifying highly variable mobile genetic elements, such as prophages, which are increasingly recognized for their role in bacterial adaptation and virulence.

In this study, we developed and applied GrafGen to a global dataset of over 1,000 *H. pylori* genomes and 366 prophages to classify strains into well-defined populations, enabling stable robust comparisons and generalizable inferences about disease associations.

## Implementation

### H. pylori genomic dataset

Training data were obtained from the *H. pylori* Genome Project (*Hp*GP), a global survey of 1011 *H. pylori* disease and control strains collected across 50 countries [3]. To evaluate the derived classification, sequences for 17 previously published ancestries were obtained from GenBank (n = 178), EnteroBase (n = 58) and Dryad (n = 19), as listed in Thorell 2023, Supplementary Table 2 [3]. Single-nucleotide polymorphisms (SNPs) were defined relative to the sequence for strain 26,695, which is a reference genome used in multiple studies, based on the most recent assembly (NCBI accession no. CP079087) [1, 3]. The genotypes included *S* = 143,733 core biallelic SNP loci with minor allele frequencies (MAFs) ≥ 1% in the *Hp*GP strains.

### Algorithm

The classification algorithm and software were adapted from the GrafPop program for human ancestry [6, 16]. The estimated phylogeographic populations and ancestral proportions were based on calculated genetic distances (GDs) as defined by Eq. (2) in Jin 2019[6]

1 $${D}_{ij}=-\frac{1}{S}{\sum }_{l\text{=}1}^{S}\left[{g}_{il}\text{ln}\left({p}_{jl}\right)\text{+}\text{ (}2 -{ g}_{jl}\text{)}\text{ln}(1 -{ p}_{jl}) \text{+}{ g}_{il}(2 -{ g}_{il})\text{ln}(2)\right]$$

where *g*_*il*_ represents the allele at SNP locus *l* in genome *i*, and *p*_*jl*_ represents the frequency of the reference allele at that SNP in population *j*.

This equation was adapted to account for the haploid nature of *H. pylori* genotypes:

$${D}_{ij}=-\frac{1}{S}{\sum }_{l\text{=}1}^{S}\left[{g}_{il}\text{ln}{(p}_{jl}) \text{+}\text{ (}1 -{ g}_{il}\text{)}\text{ln}(1 -{ p}_{jl})\right]$$

For each sample, GrafGen calculates three GD scores, GD1, GD2, and GD3, by comparing its SNP genotypes with allele frequencies of three reference populations with known ancestries, i.e., *Hp*GP European (E), African (F) and Asian (A). The GD4 value of GrafPop is not calculated by GrafGen due to lack of reference genotypes with South Asian and Latin American ancestries.

A set (denoted as *T*) of *S* = 143,733 bialllelic SNPs were selected for ancestry inference. For each reference population E, F, A, the allele frequences of all the SNPs in *T* were precalculated. Allele frequencies of 0 in a given subset were imputed as one-half the overall frequency or one-half of the inverse of the number of subset members, whichever was smaller. Given a genome *i*, as long as there are 100 or more SNPs in *T* genotyped, GrafGen runs the following steps to calculate the GD scores, and estimates ancestry proportions, like in GrafPop:

1. From genome *i*, finds all SNPs with valid genotypes, denoted as *V*, and its intersection *U* with *T*: U = V  ∩ T. Denote *N*_*U*_ as the number of SNPs in *U*.
2. Calculates the genetic distance *D*_*ij*_ from *i* to each reference population *j* using Eq. (1), considering only the *N*_*U*_ SNPs in set *U*.
3. Represents the three *D*_*ij*_ values calculated from three reference populations with a point in 3D space, denoted as *Q*_*i*_.
4. From each population *k* ∈ *{E*, *F*, *A}*, calculates the expected average value of genetic distances from every sample in *k* to each reference *j*, using the following equation that was slightly adapted from Eq. (3) in Jin 2019:
  $${E}_{D\text{\_}kj} \, \text{=} \, \frac{1}{{N}_{U}}{\sum }_{l\in U}\left[{p}_{kl}\text{ln}\left({p}_{jl}\right) \, \text{+} \, \left(1 -{ p}_{kl}\right)\text{ln}\left(1 -{ p}_{jl}\right)\right]$$

where *p*_*kl*_ is allele frequency of SNP locus *l* in population *k*. This step generates nine expected genetic distance values, represented with three 3D points, denoted as *Q*_*E*_*, Q*_*F*_*, Q*_*A*_,

5. Transforms the above four points so that *Q*_*E*_*, Q*_*F*_*, Q*_*A*_ are in *xy* plane (z = 0) of the Cartesian coordinate system. Denotes the *x, y, z* coordinates of *Q*_*i*_*,* as GD1, GD2, and GD3, respectively.
6. Calculates barycentric coordinates of GD1 and GD2 of *D*_*i*_, using *Q*_*E*_*, Q*_*F*_*, Q*_*A*_ as the three vertices of the reference triangle.
7. Calculates the expected average value of genetic distances when all of the *S* = 143,733 are considered using method similar to Step 5:
  $${E}_{D0\text{\_}kj} \, \text{=} \, \frac{1}{S}{\sum }_{l\in T}\left[{p}_{kl}\text{ln}\left({p}_{jl}\right) \, \text{+} \, \left(1 -{ p}_{kl}\right)\text{ln}\left(1 -{ p}_{jl}\right)\right]$$

This step generates three 3D points, denoted as *Q*_*E0*_*, Q*_*F0*_*, Q*_*A0*_,

8. Converts the barycentric coordinates back to Cartesian coordinates, using the triangle of *Q*_*E0*_*, Q*_*F0*_*, Q*_*A0*_ as the reference. These scores are normalized and final GD1 and GD2 values calculated.
9. Calculates the ancestry proportions* P*_*e*_,* P*_*f*_,* P*_*a*_, using Eq. 6 in Jin 2019.

Note that the SNPs with missing data are ignored, instead of imputed, by the algorithm, and the GD scores calculated using a subset of SNPs are normalized to the values expected when no data are missing, which makes the algorithm robust to data missingness.

Linkage disequilibrium (LD) or homologous recombination was not considered when the SNPs were selected for ancestry inference, since the *H. pylori* genome is small and available SNPs are limited. If LD is a concern, users can pre-prune the SNPs in LD before running GrafGen.

One of the main differences between GrafPop and GrafGen lies in how the reference population is assigned. GrafPop uses linear and parabolic relationships between normalized genetic distances (GD1–GD4) to predict the reference population for each sample (see Fig. 3 in Y. Jin et al. [6]). In contrast, GrafGen adopts a simpler approach by assigning each sample to the reference population with the minimum genetic distance. Computer outputs incorporate visualization tools that provide natural geometric interpretations of population structure. The 95% confidence bounds for distributions of each population’s normalized genetic distance scores were calculated using the ggplot2 function stat_ellipse, displayed as shaded ellipses [17].

### H. pylori population assignment

To identify population groups, we used the snippy-core function of the Snippy software tool [18] to generate a core SNP VCF file of the 1,011 *Hp*GP sequences. An initial principal component analysis (PCA) revealed that four strains from South Africa were highly divergent, obscuring differences among the rest of the sample set. We excluded those four samples as an outgroup and reran PCA on the remaining 1007 sequences. Multidimensional scaling-based k-means clustering was applied to the resulting principal components [19]. The number of clusters was chosen to be eight because adding one or two more clusters only subdivided one of these eight, thus establishing a total of nine population clusters (including the outgroup).

We calculated genetic distances from each sample to the centroid of each population cluster. For all but 7 of the 1011 sequences, the closest centroid was identical to the cluster to which it belonged. These initial clusters were refined by reassigning samples to the nearest population clusters and recalculating centroids iteratively. This process was repeated until all 1011 samples were closest to their respective population centroids, after a total of three iterations. These nine mutually exclusive assignments formed our final reference populations. The robustness of the classification to missing data was assessed by repeating population assignments using randomly selected subsets of SNPs.

Unsupervised and supervised machine learning analyses were conducted to explore the associations of SNPs with specific population groups. To initially reduce computational demands, we retained only the SNPs that had an MAF ≥ 5%, resulting in 76,066 total SNPs. While PCA is commonly used for dimensionality reduction, its linear framework limits its ability to capture complex genomic patterns. In contrast, uniform manifold approximation and projection (UMAP) applies nonlinear transformations that balance preserving both local and global structures, making it more effective for identifying subgroups within high-dimensional data [20–22]. We subsequently implemented a two-dimensional UMAP [23]. Outliers were identified and excluded if they fell below the 1st percentile or above the 99th percentile in either dimension of the projection. This UMAP and outlier removal process was iterated five times, each with a different random seed, to account for the algorithm's inherent stochasticity. On average, 73,326 SNPs (standard deviation [SD] = 681) remained across these iterations.

By examining SNPs that consistently coclustered across multiple iterations, we improved clustering robustness and minimized algorithm-driven variability. We then applied a Gaussian mixture model (GMM) to classify the filtered SNPs into clusters, with the number of clusters predefined to range from one to ten [24]. A GMM was chosen because of its flexibility in modeling clusters with diverse covariance structures. Within each cluster configuration, the proportions of minor alleles were calculated.

To determine the optimal number of SNP clusters, we used minor allele proportions from various GMM-generated SNP cluster configurations as predictors in a series of light gradient boosting machine (LightGBM) models [25]. The LightGBM was selected because of its efficiency with high-dimensional data, ability to handle categorical inputs, and relatively simple model interpretability. These qualities make it suitable for evaluating the predictive power of SNP clusters without overfitting. These models aimed to predict the nine previously defined reference populations. For each sample, the predicted class corresponded to the reference population with the highest predicted probability among the nine classes. Each LightGBM model had hyperparameters fixed at a maximum of three leaves and a maximum tree depth of three. The dataset was split, with 75% of each reference population class used for training and the remaining 25% for testing. The classification accuracy was assessed across different SNP cluster numbers, with separate LightGBM modeling performed for each of the five UMAP-filtered SNP sets. We further evaluated the model’s performance from the SNP cluster count with the highest classification accuracy using accuracy, F1 score, area under the ROC curve (AUC), sensitivity, and specificity.

Correlation analysis was conducted between the minor allele proportions of each optimal SNP cluster and the three ancestries. Due to the piecewise linear relationships between these variables, segmented models were employed to estimate breakpoints, allowing the division of minor allele proportions for fitting two separate linear models. The Pearson correlation coefficient was reported from the region exhibiting a high correlation in minor allele proportions. Because GMM cluster indices are arbitrary, we labeled them for consistency: in the case of utilizing three clusters, C1 corresponded to the European-correlated cluster, C2 to the Asian-correlated cluster, and C3 to the African-correlated cluster.

### Prophage population assignment

We separately examined the N = 366 prophage DNA sequences found in some of the 1011 *Hp*GP genomes, including n = 96 complete and n = 270 incomplete prophages. For this analysis, SNPs were defined relative to the 26,215 bp reference sequence for the bacteriophage KHP30 [26]. KHP30 was selected as the reference because it is one of the few *H. pylori* prophages with experimental evidence supporting its ability to undergo a lytic cycle [27]. Additionally, *H. pylori* prophages generally exhibit high synteny, meaning that their gene order is well conserved across different strains [4, 28]. This conservation allows for meaningful alignment against a single reference genome. While aligning to a single reference may limit the detection of certain structural variations, such as large insertions or inversions, it was necessary to ensure comparability across the dataset, especially when analyzing incomplete or rearranged prophage sequences. This approach enabled consistent SNP calling and population assignment using GrafGen. Prophage sequences were aligned to the reference prophage KHP30 using MAFFT version 7 [29], with the options –6merpair –keeplength –addfragments, to maintain the numbering of reference genome sites. The high degree of prophage genome synteny [4, 28] allows them to be aligned against a reference genome. To ensure that large gaps are not present in the alignment, the option to retain the reference genome length should be chosen, and it was selected in this analysis. The vcf file was produced from multiple sequence alignment using SNP sites [30]. Due to the high diversity of prophage sequences, nearly all SNPs are multiallelic. For GrafGen analysis, only polymorphic sites present in at least 90% of the complete prophage sequences were considered. The allele present in the majority of the reference population was treated as ancestral, whereas any alternative allele in other samples was considered to be variant.

We initially classified the 80 complete prophage sequences with conserved synteny by fineSTRUCTURE, excluding identical prophage sequences and those with large genome inversions or cargo genes that hinder alignment [9]. The classification assigned n = 12 to hpAfrica1, n = 12 to hpEastAsia, n = 28 to hpSWEurope and n = 28 to hpNEurope. The first three groups were considered vertex populations, and the fourth group represented admixture. The calculated genetic distances to the centroids of each of the four population clusters were identical to the fineSTRUCTURE assignment for 79 of the 80 sequences; this last sequence was reassigned to the closer population and centroids recalculated. The 80 samples were then confirmed to be closest to their respective population centroids, which were defined as the final training set prophage designations for GrafGen. To evaluate the derived prophage classification, a test set of 28 complete prophage sequences previously classified by fineSTRUCTURE was used [15].

All analyses were conducted via R version 4.3.2, with the UMAP, GMM, and LightGBM methods implemented via the CRAN packages *umap*, *mclust*, and *lightgbm*, respectively.

## Results

### Population assignment and validation

**Fig. 1:**
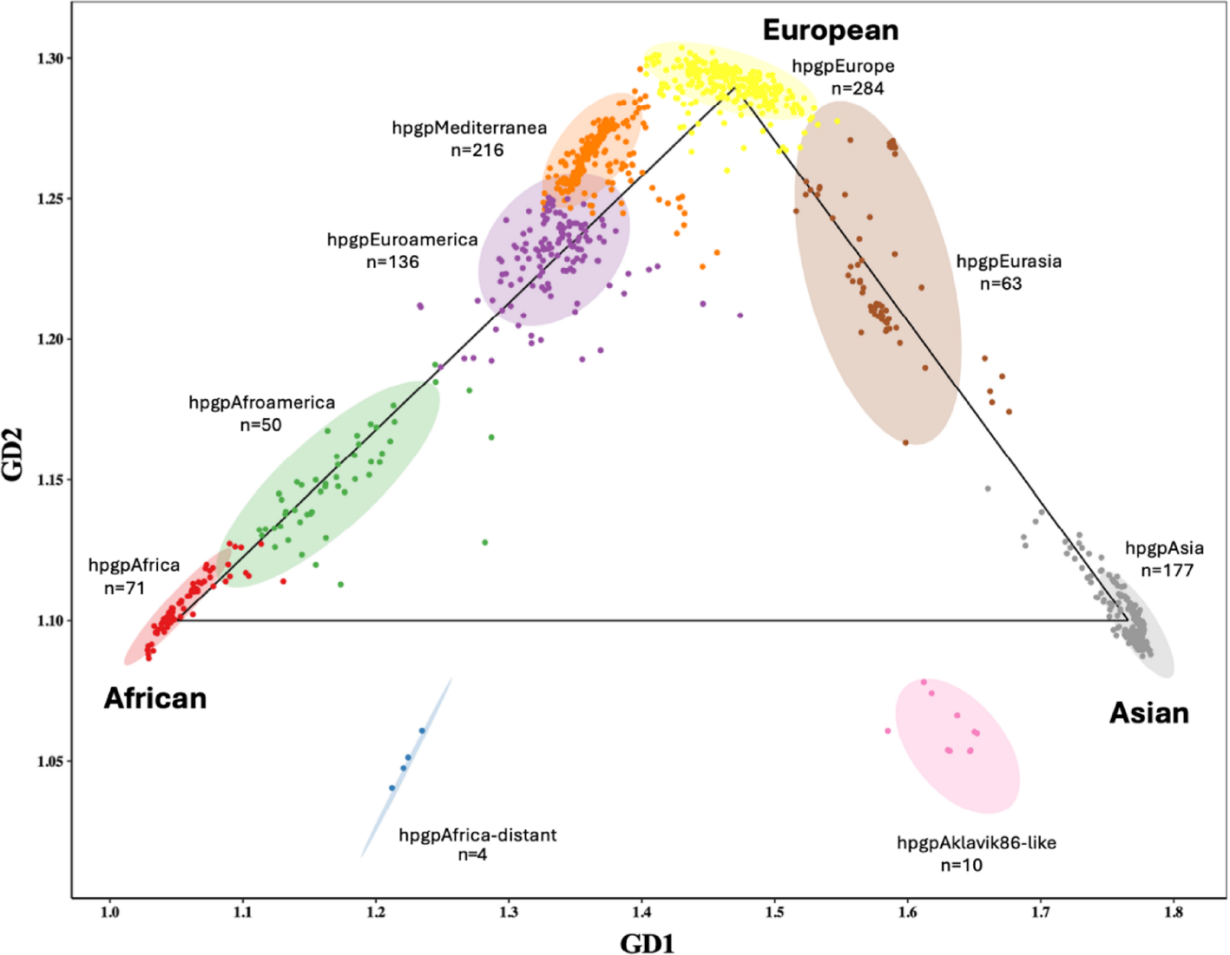
Genetic distance-based classification of 1,011 *Hp*GP sequences from 50 countries into nine phylogeographic clusters

The nine clusters of *Hp*GP sequences from 50 countries differed by predominant geographic source (Fig. 1). Three major continental ancestries were identifiable: hpgpAfrica, hpgpAsia and hpgpEurope. The four intermediate admixed groups were designated hpgpAfroamerica, hpgpEuroamerica, hpgpMediterranea and hpgpEurasia. The divergent South African samples were named hpgpAfrica-distant, and the remaining outgroup was denoted hpgpAklavik86-like [31]. Strains from individual countries were classified into one (n = 15), two (n = 16), three (n = 8), four (n = 5), five (n = 3), six (n = 2), or seven (n = 1) GrafGen groups (Supplemental Table 1). Strains from gastric cancer, metaplasia or gastritis patients in a given country tended to have similar classifications (Supplemental Fig. 1).

**Table 1:** GrafGen population assignments of 255 GenBank/other test sequences previously classified into 17 ancestry populations/subpopulations

Population/subpopulation	hpgpAfrica	hpgpAfrica-distant	hpgpAfroamerica	hpgpEuroamerica	hpgpMediterranea	hpgpEurope	hpgpEurasia	hpgpAsia	hpgpAklavik86-like	TOTAL
hspAfrica1WAfrica	15									**15**
hspAfrica1SAfrica	10		5							**15**
hpAfrica2		15								**15**
hspAfrica1LatinAmerica	6		9							**15**
hspSWEuropeLatinAmerica				11	4					**15**
hpNEAfrica				5	10					**15**
hspSWEurope				2	13					**15**
hspSEurope					3	12				**15**
hspNEurope						12	3			**15**
hspSiberia							14	1		**15**
hpAsia2							15			**15**
hspUral							15			**15**
hpNorthAsia							2	10	3	**15**
hpSahul							7	8		**15**
hspEAsia								15		**15**
hspIndigenousSAmerica							1	14		**15**
hspIndigenousNAmerica								4	11	**15**
Total	**31**	**15**	**14**	**19**	**29**	**24**	**58**	**51**	**14**	**255**

The total number for each population of the nine populations defined in GrafGen is shown in bold

**Fig. 2:**
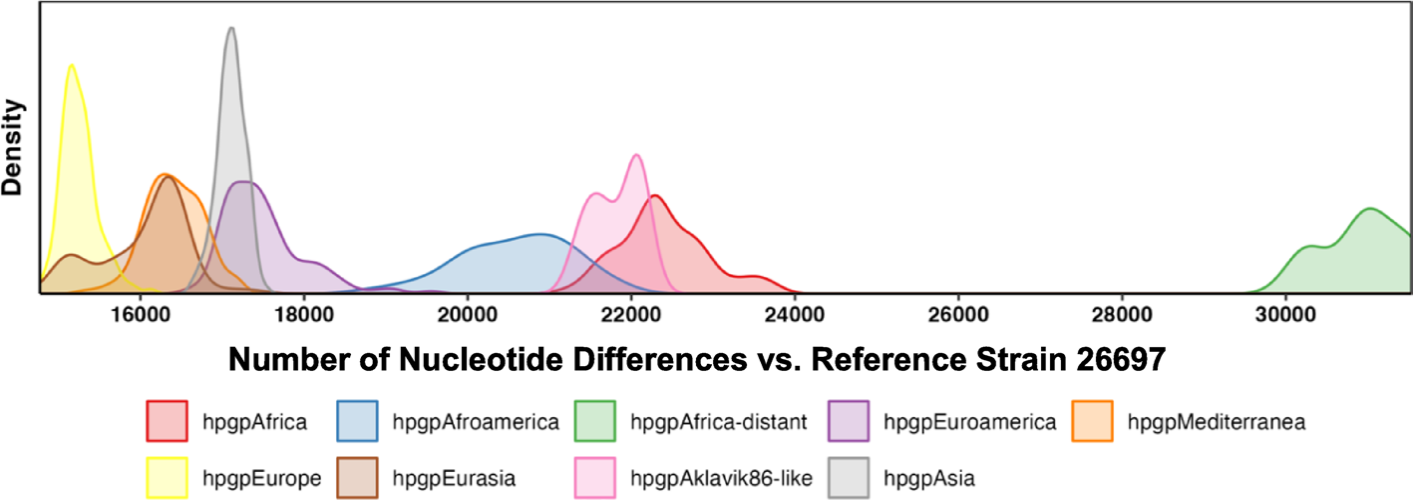
Distributions of core SNP frequencies for 1,011 *Hp*GP genomes according to the GrafGen population

The total core SNP frequency (vs. 26,695) was lowest for the hpgpEurope strains (mean ± SD, 11,583 ± 8026) and highest for the hpgpAfrica-distant strains (36,782 ± 6749; Fig. 2), which is consistent with the European source of the reference genome. This pattern reflects the phylogeographic relationship between strains and highlights the influence of reference genome ancestry on SNP counts: strains more closely related to 26,695, which is of European origin, tend to have fewer SNPs. Conversely, more divergent strains (e.g., from Africa) accumulate more SNPs due to their deeper evolutionary separation. These core SNP differences reinforce the population structure observed in the phylogenetic tree and support distinct ancestral lineages among geographic groups.

Previously published ancestries of 255 GenBank/other test sequences mapped to specific GrafGen classifications. Each set of 15 strains from a given published ancestry was classified into just one (n = 5) or two (n = 11) GrafGen groups, with the exception of hpNorthAsia, which was classified into three groups (Table 1). 

**Fig. 3:**
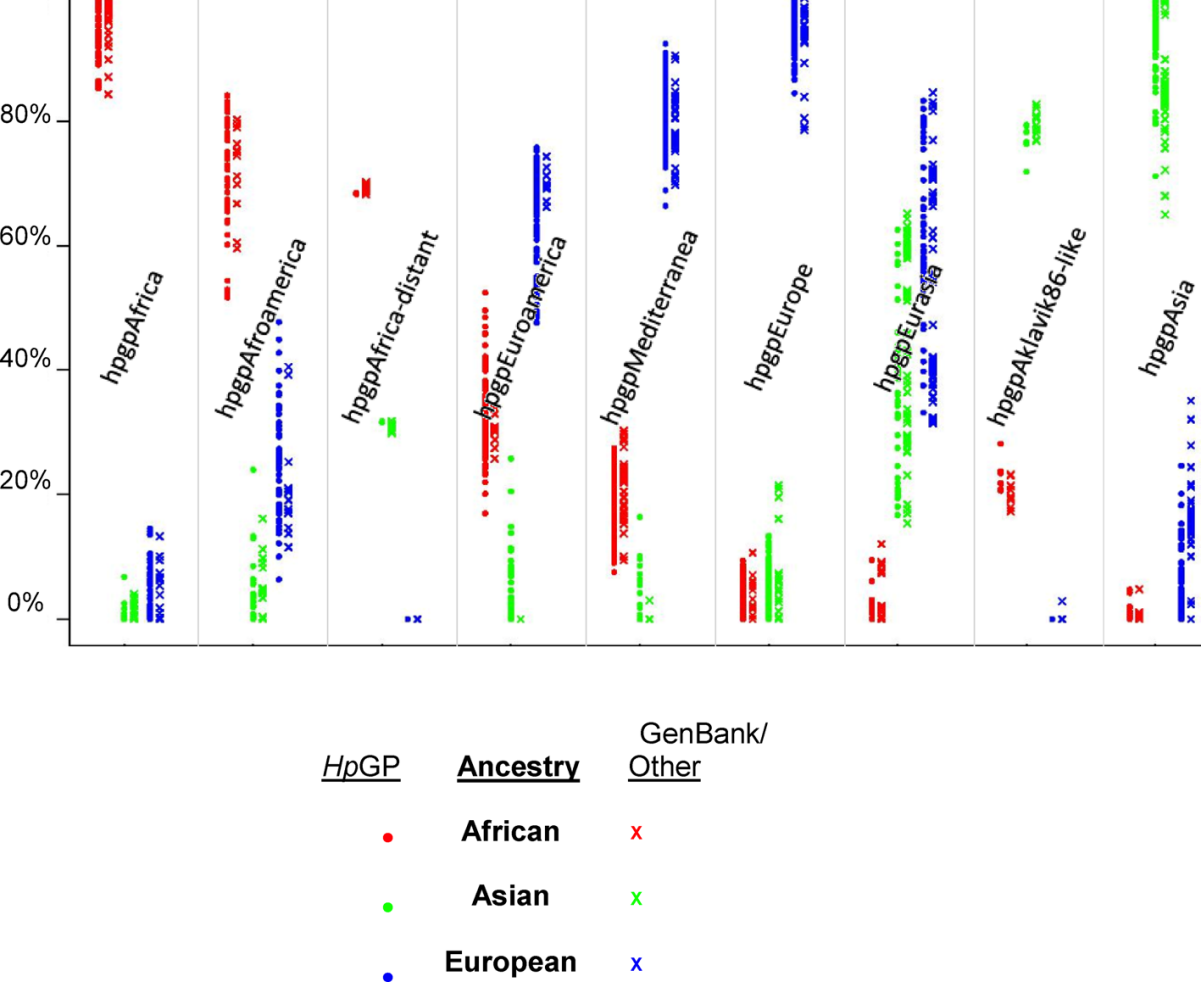
Estimated ancestral proportions of 1,011 *Hp*GP and 255 test sequences in the GrafGen population

The estimated proportions of African, Asian and European ancestry of *Hp*GP and GenBank/other strains varied according to the GrafGen classification. Going from left to right as ordered in the figure, the African proportion monotonically declined, and the Asian proportion monotonically increased, with the European proportion maximal in the middle (Fig. 3).

The LightGBM model performance across varying numbers of GMM-derived SNP clusters is presented in Supplemental Fig. 3 to identify the optimal cluster count for the final model input. Models using three and nine clusters achieved the highest accuracies (94.3% and 94.7%, respectively). Given their comparable performance, the three-cluster model was selected for greater simplicity and interpretability. Across the five runs, the LightGBM model using only three SNP clusters achieved a mean accuracy of 94.35% (SD 1.29), an F1 score of 0.93 (SD 0.01), overall AUC of 0.64 (SD 0.04), overall sensitivity of 92.27% (SD 2.26), and overall specificity of 99.25% (SD 0.18)—with AUC, sensitivity, and specificity computed as the unweighted average of per-class metrics across all nine reference populations. On the single best run, the model reached 96.08% accuracy, an F1 score of 0.92, AUC of 0.61, sensitivity of 91.61%, and specificity of 99.49%. The corresponding confusion matrix for this top-performing run is shown in Supplemental Table 2. The confusion matrix indicates perfect classification for hpgpAfrica (n = 18), hpgpAsia (n = 45), hpgpEurasia (n = 16), and hpgpEurope (n = 71). Supplemental Fig. 4 depicts predicted probabilities for the test cases, with misclassified samples’ quartiles ranging from 0.74 to 0.98 versus 0.99 to 1.00 for correct cases, indicating greater confidence in accurate predictions and lower confidence to erroneous ones. The LightGBM variable importance analysis showed that C1, C2, and C3 accounted for 28%, 33%, and 39% of the total gain, suggesting that all three clusters contributed comparably to the model’s predictive performance.

**Table 2:** GrafGen classification performance using randomly selected 10% or 1% of the SNPs

Relative performance	hpgpAfrica	hpgpAfrica-distant	hpgpAfroamerica	hpgpEuroamerica	hpgpMediterranea	hpgpEurope	hpgpEurasia	hpgpAsia	hpgpAklavik86-like
14,300 SNPs: Sensitivity	100%	100%	99%	100%	99%	99%	98%	99%	100%
Specificity	100%	100%	100%	100%	100%	100%	100%	100%	100%
1430 SNPs: Sensitivity	98%	100%	94%	94%	93%	95%	89%	98%	100%
Specificity	100%	100%	100%	99%	99%	98%	99%	99%	100%

**Fig. 4:**
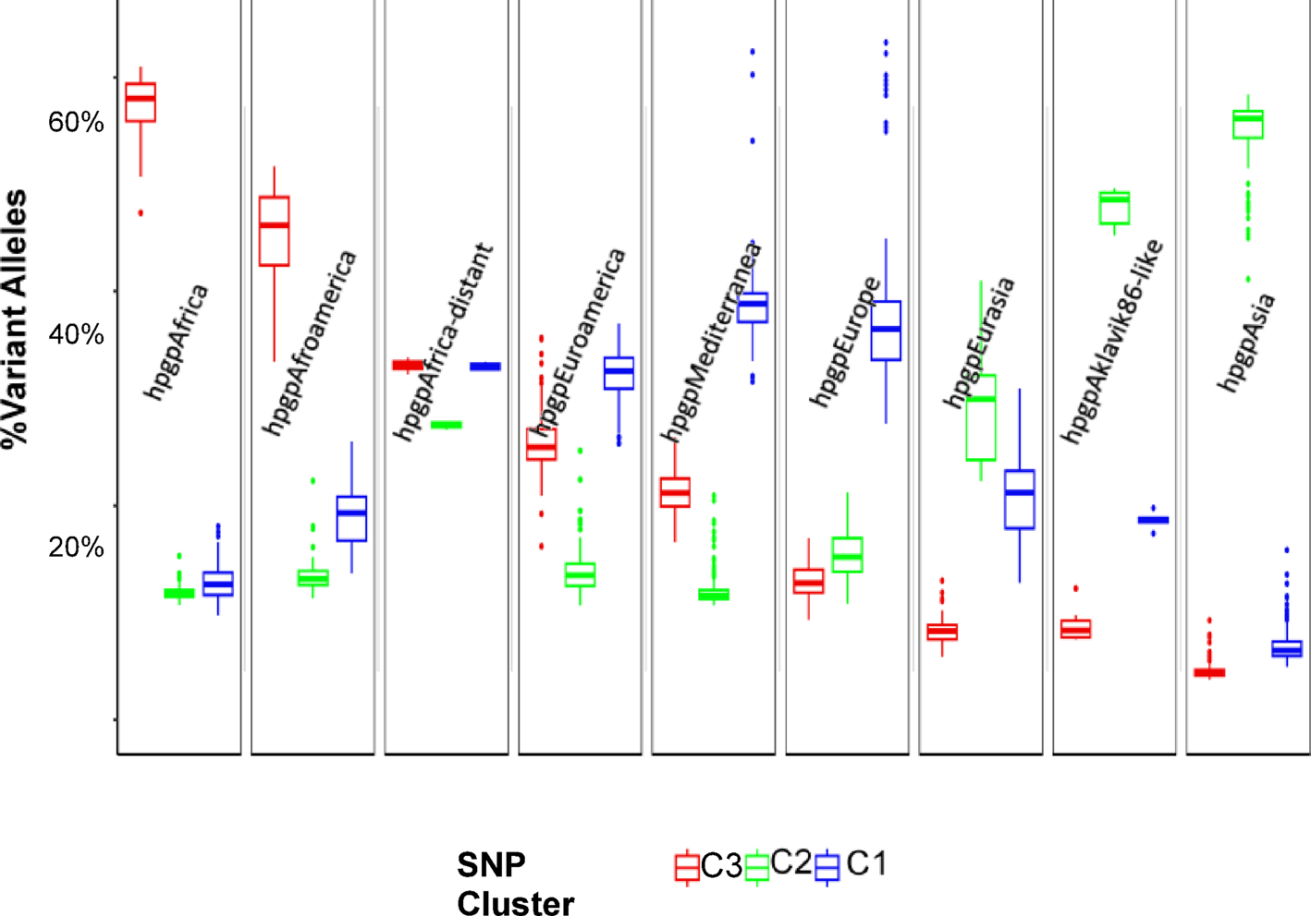
Variant allele frequencies for three GMM-identified SNP clusters of 1,011 *Hp*GP genomes in the GrafGen population

Sixty-four percent (48,396/76,066) of the SNPs were consistently assigned to a given cluster in all five GMM-identified models. These clusters contained 16 ± 11% (C1), 34 ± 3% (C2), and 50 ± 9% (C3) of the SNPs, respectively, which varied in frequency across the nine reference populations. Figure 4 further illustrates the population structure by showing the distribution of variant allele frequencies across SNP clusters (C1–C3) within each reference population group. Notably, C3 variants are highly enriched in African populations (hpgpAfrica, hpgpAfroamerica, hpgpAfrica-distant), where they often constitute more than 40–60% of variant alleles, suggesting that these SNPs are specific to ancestral African lineages. In contrast, C2 variants are most prevalent in Asian groups (e.g., hpgpAsia, hpgpEurasia, hpgpAkakvik86-like), while C1 variants dominate in European and Mediterranean populations (e.g., hpgpEurope, hpgpMediterranea). These distinct allele frequency patterns indicate limited gene flow between populations and support the presence of geographically structured SNP clusters that reflect long-term population separation. Moreover, the differential enrichment of SNP clusters provides additional support for ancestry inference, as each group shows a characteristic signature of SNP cluster composition. Together with the core SNP counts in Fig. 2, these results reveal a clear correspondence between genomic variation and phylogeographic origin. Each cluster’s minor allele proportion was strongly correlated with one of the three ancestries, with piecewise linear correlation coefficients (r) of 0.92 ± 0.02 for the “European” cluster C1, 1.00 ± 0.00 for the “Asian” cluster C2, and 1.00 ± 0.01 for the “African” cluster C3 (Supplemental Fig. 2).

Our results show that the algorithm is robust to LD or homologous recombination. Population classifications obtained using a filtered set of 75,593 SNPs with pairwise correlations less than 0.1 were > 99% identical to those obtained with all 143,705 SNPs. Population assignments based on 1000 randomly selected subsets of 10% or 1% of SNPs had 93–100% sensitivity and specificity for the gold standard classifications that used all available SNPs (Table 2). In addition, in our validation, repeating the analysis on an LD-pruned panel (PLINK2 --indep-pairwise 10 5 0.01; ${r}^{2}<0.01\approx r<0.1$) yielded near-identical results across *n* = 1011 genomes: 99.51% top-1 agreement with the full panel (Cohen’s κ = 0.9939), with mean absolute error (MAE) changes of 2.02, 3.12, and 1.51 percentage points (pp) for F, E, and A, respectively (overall MAE = 2.22 pp). Only samples in hpgpEurope and hpgpMediterranea interexchange, 0.5% from hpgpMediterranea to hpgpEurope while 1.4% from hpgpEurope to hpgpMediterranea. The rest assignments (hpgpEurasia, hpgpAsia, hpgpAklavik86-like, hpgpAfroamerica, hpgpAfrica-distant, and hpgpAfrica) are the same. These findings indicate GrafGen’s ancestry estimates are robust to LD/recombination and SNP subsampling.

### Benchmarking

To benchmark GrafGen against existing methods, we applied fastBAPS[12], fineSTRUCTURE[9], and PopPUNK[13] to the same *Hp*GP dataset. We used two popular measurements, Adjusted Rand Index (ARI, ranging from -1 to 1) and Normalized Mutual Information (NMI, ranging from 0 to 1) to evaluate the similarity between any two clustering assignments. Both have 1 for perfect match and 0 for random labeling. PopPUNK gives more than 900 clusters using either the software-provided *H. pylori* database (https://ftp.ebi.ac.uk/pub/databases/pp_dbs/Helicobacter_pylori_v1_refs.tar.bz2) or the current study dataset. The results from fastBAPS gave us 5, 7, 8, and 13 clusters at multi-level assignment. fastBAPS shows higher agreement with GrafGen(reference clustering) than fineSTRUCTURE, in terms of both ARI (0.711 vs. 0.625) and NMI (0.722 vs. 0.643). The difference is moderate, but fastBAPS appears to produce cluster assignments more consistent with GrafGen (Supplemental Table 3). In addition, GrafGen used less CPU time and physical RAM than others when tested on an Apple MacBook Pro with M1 10-core CPU (only one core was used for the work), 16 GB memory under macOS Sequoia 15.5, demonstrating its competitive performance and scalability. Sankey plot showed the cluster assignment flow across methods (Supplemental Fig. 5). The benchmarking results, code, and other documentation can be accessed at https://github.com/wangdi2016/GrafGen-manuscript.git.

GrafGen prophage assignments of 28 test sequences substantially agreed with previous fineSTRUCTURE categorizations. In particular, all 6 hpSWEurope sequences identified by fineSTRUCTURE were hpgpSWEurope by GrafGen, and all 4 hpEastAsia sequences were hpgpEastAsia; 5 of the 7 hpNEurope sequences were hpgpNEurope and 2 were hpgpEastAsia; and 8 of the 11 hpAfrica1 sequences were hpgpAfrica1 and 3 were hpgpNEurope (Cohen’s kappa = 0.76). Notably, both of the discordant hpNEurope prophages had hpEastAsia DNA fragments detectable by fineSTRUCTURE, reflecting recombination between these groups. Furthermore, the 3 discordant hpAfrica1 prophages had genetic distances to the respective *Hp*GP population centroids within 5%, which is indicative of ambiguous classification.

Among the N = 16 *Hp*GP complete prophages that could not be classified by fineSTRUCTURE due to large insertions and/or inversions, 1 was classified as hpgpSWEurope, 4 as hpgpEastAsia, 6 as hpgpNEurope and 5 as hpgpAfrica1 by GrafGen. Among the N = 270 incomplete *Hp*GP prophage sequences, 75 were classified as hpgpSWEurope, 68 as hpgpEastAsia, 85 as hpgpNEurope and 42 as hpgpAfrica1 (Supplemental Fig. 6).

## Discussion

The *H. pylori* genome is less than one-thousandth the size of the human diploid genome. With nearly 10% of core loci polymorphic, it is 100 times more diverse than the human genome [32]. While the SNP clusters strongly correlated with ancestral components, further investigation is needed to determine whether certain clusters are enriched for functional or pathogenic loci, which could enhance biological interpretation. Indeed, SNP differences may be functionally significant for carcinogenicity, warranting investigation. Importantly, population stratification is a potential source of bias since *H. pylori* genomes vary systematically within and between countries and ethnic groups. The striking similarity to the structure of the human population reflects symbiotic coevolution within infected stomachs and the two species’ common migration histories [33]. Adjustment for ancestry is thus necessary to control confounding in bacterial GWAS for generalizable, valid inferences. GWAS is intended for identifying associations between genetic variants and traits across populations. However, in the case of *H. pylori*, due to the high level of phylogeographic signal and structured populations, the analysis may capture this signal rather than true GWAS associations. This underscores the importance of selecting a robust group of genomes from the same population for a meaningful GWAS analysis.

GrafGen is an open-source R package designed for *H. pylori* genomics and ancestry adjustment in GWAS. Similarly to GrafPop, the algorithm is robust to large amounts of missing data, facilitating comparisons among multiple genotype datasets [6]. The tool also performs *H. pylori* prophage classification, which may be particularly useful for incomplete prophages and/or disrupted synteny; previous typing of such sequences was necessarily limited to the two conserved genes, holin and integrase [4, 34, 35]. Therefore, while GrafGen does not directly produce an output to be used in GWAS, it provides valuable insights that allow researchers to make informed decisions about population structure. By classifying genomes effectively, GrafGen enables researchers to assess the data better and ensure that the right populations are used in GWAS. Furthermore, the GrafGen algorithm is potentially extendable to any haploid species with sufficient reference data. Other candidates for microbial GWAS that may require control of potential population stratification include the Epstein–Barr virus, *Plasmodium falciparum*, human papillomavirus 16 and hepatitis C virus.

While GrafGen was adapted from a method originally developed for human population analysis, its application to *H. pylori* presents unique challenges. Bacterial genomes, especially those of highly recombinogenic bacteria with considerable genome plasticity, such as *H. pylori* [14, 15], can complicate ancestry inference. However, one of the key advantages of GrafGen is that it provides stable and consistent population classification, in contrast to many existing bacterial methods whose results can vary depending on the specific set of strains included in the analysis. By using genetic distances to reference populations derived from a large and diverse global dataset, GrafGen enables robust and reproducible classification of both complete and partial genomes, even across studies. This stability is critical for comparative and association studies, ensuring that population assignments remain consistent regardless of dataset composition. The small number of misclassifications primarily involved admixed genomes. These ambiguous cases highlight the importance of incorporating ancestral proportions rather than categorical labels alone in downstream analyses.

The stable population structure observed in *H. pylori* genomes using GrafGen offers a robust framework for tracing human migration patterns, given the long-standing coevolution between *H. pylori* and its human hosts [36, 37]. Similarly, GrafGen performs well in classifying prophage populations, which is biologically meaningful because prophages play key roles in horizontal gene transfer, bacterial evolution, and the modulation of virulence [28, 38]. By reliably assigning prophages to distinct population groups, GrafGen enables the study of phage–host coevolution and the potential contribution of prophage diversity to bacterial adaptation and pathogenic potential. GrafGen’s stability and flexibility make it a promising tool for additional applications, including real-time tracking of bacterial evolution, detection of recent admixture events, and integration with temporal or spatial metadata in outbreak investigations.

In addition, our benchmarking results show that GrafGen offers a favorable balance of accuracy and efficiency compared to established tools. fineSTRUCTURE is very computationally intensive. This restricts its utility for very large datasets, e.g. 10,000 samples. Unlike fastBAPS, which may struggle with very large datasets in terms of memory, and PopPUNK, which relies on k-mer distances and in our benchmark generating too many single-sample clusters, GrafGen leverages sparse SNP data for interpretable and scalable clustering.

One of the key limitations of GrafGen is its reliance on a predefined reference panel and comparisons to a single standard genome (strain 26,695). These features may hinder classification accuracy for novel or undersampled lineages, particularly in regions with limited genomic surveillance. Another limitation of GrafGen is that it employs a distance-based clustering approach without explicitly modeling phylogenetic relationships or homologous recombination. This choice improves computational scalability and makes the method robust to incomplete or fragmented genomes, but it may overlook subtle signals of shared ancestry or reticulate evolution. This limitation is particularly relevant for highly recombinogenic organisms such as *H. pylori*, where recombination can obscure tree-like relationships. However, this limitation seemingly does not hinder the correct identification of the unique hardy strain type that we recently discovered, amongst ubiquitous-type strains (Supplemental Table 2, Supplemental Figs. 5 and 7)[39].

For applying GrafGen to a new bacterial species, we recommend building reference populations from an independent panel of isolates spanning geography and host. The de novo genomes are sequenced on the same or similar platform like PacBio long reads sequence platform Sequel/SequelII to avoid batch effect which would put a bias on the ancestry assignment. In terms of sample size, we suggest more than 100 highly diverse de novo genomes.

GrafGen is freely available for use by the scientific community studying *H. pylori* and for adaptation to other microbial genomes from https://bioconductor.org/packages/GrafGen and https://github.com/wheelerb/GrafGen/tree/v2.0_beta.

## Conclusions

We developed GrafGen, an open-source R package for robust classification of *Helicobacter pylori* ancestry and prophage diversity. By leveraging global reference data and a genetic distance–based framework, GrafGen provides consistent population assignments even with reduced SNP sets, overcoming limitations of prior model- and distance-based methods. The tool enables reliable correction for population stratification in bacterial GWAS, thereby improving the accuracy of genotype–phenotype associations. Integrated visualization features offer intuitive interpretation of ancestry relationships, enhancing usability for comparative and evolutionary studies. Importantly, GrafGen is not restricted to *H. pylori*; the underlying algorithm is readily extensible to other haploid organisms with suitable reference datasets. As such, GrafGen represents a broadly applicable resource for microbial population genomics and the study of host–pathogen interactions.

### Availability and requirements

Project name: GrafGen. Project home page: https://github.com/wheelerb/GrafGen. Operating system(s): Platform independent. Programming language: C ++ /C, R. License: MIT. Any restrictions to use by non-academics: None.

## Supplementary Information

Below is the link to the Supplementary Information.

Supplementary Material 1

## Data Availability

The open-source R package (version 1.4.0) for the analysis of *H. pylori* genomes with a fixed set of nine reference populations is included in Bioconductor (https://bioconductor.org/packages/GrafGen). A more general version of the package (v2.0_beta) for analyzing multichromosome haploid genomes with varying reference populations as well as *H. pylori* prophages is available on GitHub (https://github.com/wheelerb/GrafGen/tree/v2.0_beta). Previous published *H. pylori* genome sequence data can be access at 10.5281/zenodo.10048320. The benchmark data, results, and code can be found at https://github.com/wangdi2016/GrafGen-manuscript.git.

## Abbreviations

SNP: Single nucleotide polymorphism
GD: Genetic distance
UMAP: Uniform manifold approximation and projection
PCA: Principal component analysis
VCF: Variant call format
MAF: Minor allele frequency
GMM: Gaussian mixture model
LightGBM: Light gradient boosting machine
*H. pylori*: *Helicobacter pylori*
*Hp*GP: *H. pylori* Genome Project
GWAS: Genome-wide association study
