# Supplementary material for: GrafGen: distance-based inference of population ancestry for Helicobacter pylori genomes
Source: BMC Bioinformatics. 2025 Nov 26;26:308. doi: 10.1186/s12859-025-06294-y (PMC12751694; doi:10.1186/s12859-025-06294-y)
Supplement: Supplementary file 1 — Supplementary Material 1 [file 12859_2025_6294_MOESM1_ESM.docx]

Supplemental Table 1. Generic classification of 1,011 *Hp*GP sequences from 50 countries

| **COUNTRY** | **hpgpAfrica** | **hpgpAfrica-distant** | **hpgpAfroamerica** | **hpgpEuroamerica** | **hpgpMediterranea** | **hpgpEurope** | **hpgpEurasia** | **hpgpAsia** | **hpgpAklavik86-like** | **TOTAL** |
| --- | --- | --- | --- | --- | --- | --- | --- | --- | --- | --- |
| Algeria | 0 | 0 | 0 | 2 | 8 | 0 | 0 | 0 | 0 | **10** |
| Argentina | 0 | 0 | 1 | 1 | 5 | 0 | 0 | 3 | 0 | **10** |
| Bangladesh | 0 | 0 | 0 | 0 | 0 | 6 | 4 | 0 | 0 | **10** |
| Brazil | 10 | 0 | 2 | 2 | 4 | 2 | 0 | 1 | 0 | **21** |
| Bulgaria | 0 | 0 | 0 | 0 | 0 | 8 | 0 | 0 | 0 | **8** |
| Canada | 0 | 0 | 3 | 0 | 0 | 0 | 6 | 3 | 8 | **20** |
| Chile | 0 | 0 | 3 | 18 | 22 | 0 | 0 | 1 | 2 | **46** |
| China | 0 | 0 | 0 | 0 | 0 | 0 | 0 | 10 | 0 | **10** |
| Colombia | 2 | 0 | 8 | 23 | 12 | 0 | 0 | 0 | 0 | **45** |
| Costa Rica | 0 | 0 | 2 | 6 | 0 | 0 | 0 | 0 | 0 | **8** |
| Dominican Republic | 3 | 0 | 1 | 2 | 5 | 0 | 0 | 0 | 0 | **11** |
| DR Congo | 10 | 0 | 0 | 0 | 1 | 0 | 0 | 0 | 0 | **11** |
| France | 0 | 0 | 0 | 1 | 9 | 11 | 0 | 0 | 0 | **21** |
| Germany | 0 | 0 | 0 | 0 | 1 | 16 | 0 | 0 | 0 | **17** |
| Ghana | 2 | 0 | 0 | 0 | 0 | 0 | 0 | 0 | 0 | **2** |
| Greece | 0 | 0 | 0 | 0 | 1 | 20 | 0 | 0 | 0 | **21** |
| Guatemala | 0 | 0 | 1 | 1 | 1 | 0 | 0 | 0 | 0 | **3** |
| Honduras | 1 | 0 | 13 | 11 | 1 | 0 | 0 | 0 | 0 | **26** |
| Iceland | 0 | 0 | 0 | 0 | 0 | 0 | 11 | 0 | 0 | **11** |
| India | 0 | 0 | 0 | 0 | 0 | 5 | 5 | 0 | 0 | **10** |
| Indonesia | 0 | 0 | 0 | 1 | 2 | 2 | 2 | 4 | 0 | **11** |
| Iran | 0 | 0 | 0 | 0 | 0 | 4 | 0 | 0 | 0 | **4** |
| Israel | 0 | 0 | 0 | 0 | 4 | 6 | 0 | 0 | 0 | **10** |
| Italy | 0 | 0 | 0 | 0 | 3 | 26 | 0 | 0 | 0 | **29** |
| Japan | 0 | 0 | 0 | 1 | 0 | 0 | 2 | 26 | 0 | **29** |
| Jordan | 0 | 0 | 0 | 0 | 4 | 6 | 0 | 0 | 0 | **10** |
| Kazakhstan | 0 | 0 | 0 | 0 | 0 | 1 | 1 | 0 | 0 | **2** |
| Kyrgyzistan | 0 | 0 | 0 | 0 | 0 | 5 | 5 | 0 | 0 | **10** |
| Latvia | 0 | 0 | 0 | 0 | 0 | 34 | 0 | 0 | 0 | **34** |
| Lithuania | 0 | 0 | 0 | 0 | 0 | 23 | 0 | 0 | 0 | **23** |
| Malaysia | 0 | 0 | 0 | 0 | 1 | 0 | 6 | 12 | 0 | **19** |
| Mexico | 0 | 0 | 0 | 17 | 4 | 0 | 0 | 1 | 0 | **22** |
| Myanmar | 0 | 0 | 0 | 0 | 0 | 0 | 12 | 0 | 0 | **12** |
| Nepal | 0 | 0 | 0 | 0 | 0 | 7 | 6 | 0 | 0 | **13** |
| Nigeria | 2 | 0 | 0 | 2 | 0 | 0 | 0 | 0 | 0 | **4** |
| Peru | 1 | 0 | 4 | 22 | 1 | 0 | 0 | 5 | 0 | **33** |
| Poland | 0 | 0 | 0 | 0 | 0 | 20 | 0 | 0 | 0 | **20** |
| Portugal | 2 | 0 | 0 | 4 | 24 | 0 | 0 | 0 | 0 | **30** |
| Russian Federation | 0 | 0 | 0 | 0 | 0 | 10 | 0 | 0 | 0 | **10** |
| Singapore | 0 | 0 | 0 | 0 | 0 | 0 | 0 | 21 | 0 | **21** |
| South Africa | 4 | 4 | 0 | 1 | 0 | 0 | 0 | 0 | 0 | **9** |
| South Korea | 0 | 0 | 0 | 0 | 0 | 0 | 1 | 53 | 0 | **54** |
| Spain | 3 | 0 | 2 | 13 | 83 | 4 | 0 | 1 | 0 | **106** |
| Sweden | 0 | 0 | 0 | 0 | 0 | 29 | 1 | 0 | 0 | **30** |
| Switzerland | 0 | 0 | 0 | 1 | 6 | 8 | 0 | 0 | 0 | **15** |
| Taiwan | 0 | 0 | 0 | 0 | 0 | 0 | 0 | 24 | 0 | **24** |
| The Gambia | 5 | 0 | 0 | 0 | 0 | 0 | 0 | 0 | 0 | **5** |
| Türkiye | 0 | 0 | 0 | 0 | 0 | 17 | 0 | 0 | 0 | **17** |
| USA | 26 | 0 | 10 | 7 | 14 | 14 | 1 | 3 | 0 | **75** |
| Vietnam | 0 | 0 | 0 | 0 | 0 | 0 | 0 | 9 | 0 | **9** |
| **TOTAL** | **71** | **4** | **50** | **136** | **216** | **284** | **63** | **177** | **10** | **1011** |

Supplemental Table 2. Confusion matrix for the test set from the LightGBM model

|  |  | **Actual Refpop** | | | | | | | | |
| --- | --- | --- | --- | --- | --- | --- | --- | --- | --- | --- |
|  |  | hpgpAfrica | hpgpAfrica-distant | hpgpAfroamerica | hpgpAklavik86-like | hpgpAsia | hpgpEurasia | hpgpEuroamerica | hpgpEurope | hpgpMediterranea |
| **Refpop Prediction** | hpgpAfrica | 18 | 0 | 3 | 0 | 0 | 0 | 0 | 0 | 0 |
|  | hpgpAfrica-distant | 0 | 1 | 0 | 0 | 0 | 0 | 0 | 0 | 0 |
|  | hpgpAfroamerica | 0 | 0 | 9 | 0 | 0 | 0 | 0 | 0 | 0 |
|  | hpgpAklavik86-like | 0 | 0 | 0 | 2 | 0 | 0 | 0 | 0 | 0 |
|  | hpgpAsia | 0 | 0 | 0 | 0 | 45 | 0 | 0 | 0 | 0 |
|  | hpgpEurasia | 0 | 0 | 0 | 1 | 0 | 16 | 0 | 0 | 0 |
|  | hpgpEuroamerica | 0 | 0 | 1 | 0 | 0 | 0 | 32 | 0 | 2 |
|  | hpgpEurope | 0 | 0 | 0 | 0 | 0 | 0 | 0 | 71 | 1 |
|  | hpgpMediterranea | 0 | 0 | 0 | 0 | 0 | 0 | 2 | 0 | 51 |

Note: The nine reference populations from the 255 test samples were predicted using the three minor-allele proportion clusters identified by the Gaussian mixture model.

Supplemental Table 3. Comparison of GrafGen with other population classification methods

| Method | No. clusters | ARI^*^ | NMI^*^ | Version | CPU Time(s) | No. Core | RAM(GB) |
| --- | --- | --- | --- | --- | --- | --- | --- |
| GrafGen | 9 | 1.000 | 1.000 | 2.0_beta | 27.1 | 1 | 0.7 |
| fastBAPS | 5  7  8  13 | 0.673  0.711  0.711  0.711 | 0.672  0.722  0.722  0.722 | 1.0.8 | 193.8 | 1 | 3.0 |
| fineSTRUCTURE^#^ | 4  13 | 0.647  0.625 | 0.617  0.643 | 4.0 | > 10 days | 1 | 256 |
| PopPUNK | 985 | 0.001 | 0.267 | 2.7.6 | 147.8 | 1 | 0.6 |

# Our previously published classification results from *Hp*GP [3]. The running time and RAM are estimated [Zilia Yanira Munoz Ramirez, personal communication]

* Both ARI and NMI stand for Adjusted Rand Index and Normalized Mutual Information, which are two commonly used metrics to evaluate the similarity between two clusterings.

For GrafGen, fastBAPS, and PopPUNK, the running test was carried out on Apple MacBook Pro with M1 10-core CPU, 16 GB memory under macOS Sequoia 15.5.

Supplemental Figure 1. Genetic distance-based clustering of *Hp*GP sequences from 233 patients with gastric cancer (panel **A**), 172 patients with advanced intestinal metaplasia (panel **B**), and 606 patients with nonatrophic gastritis (panel **C**). Color assigned by nine clusters.


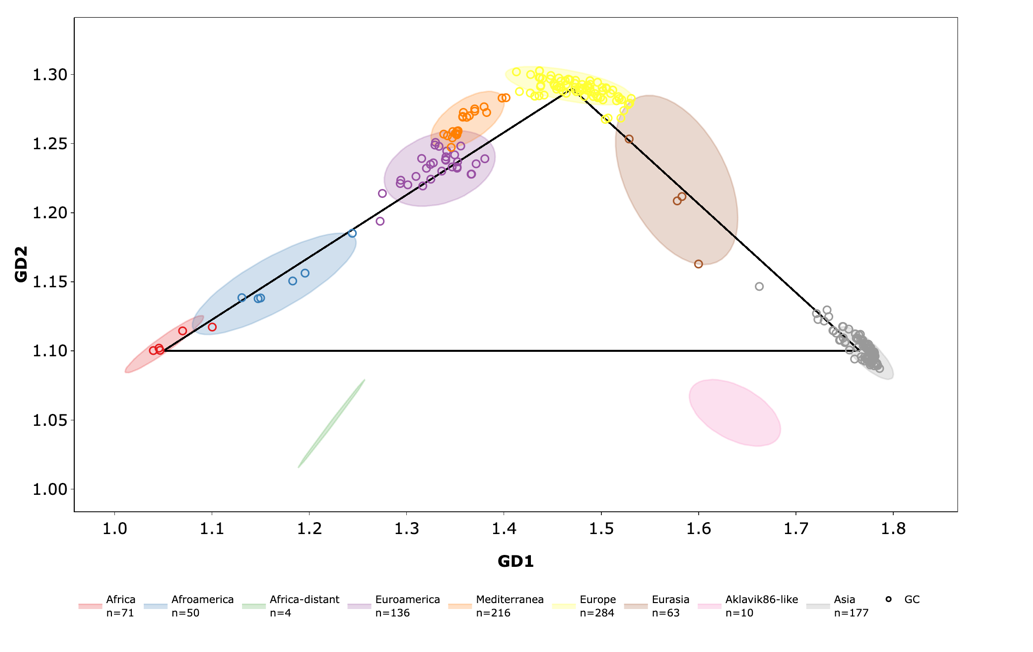


hpgpAfroamerica

hpgpEuroamerica

**Asian**

hpgpEurasia

hpgpMediterranea

hpgpAfrica-distant

hpgpAfrica

**African**

hpgpAklavik86-like

**European**

hpgpEurope

hpgpAsia

**A**


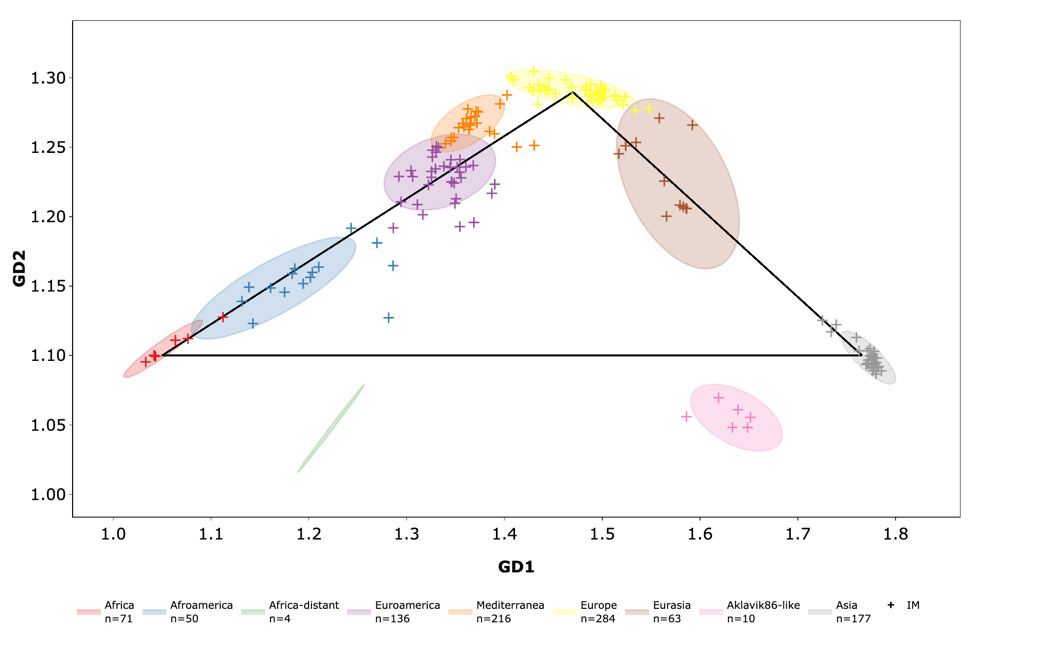

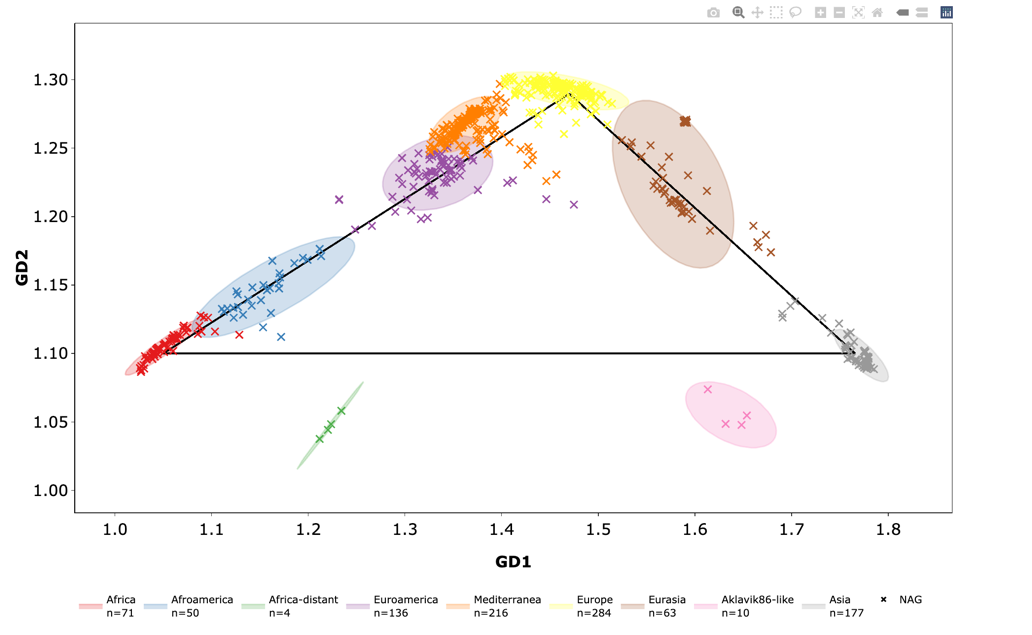

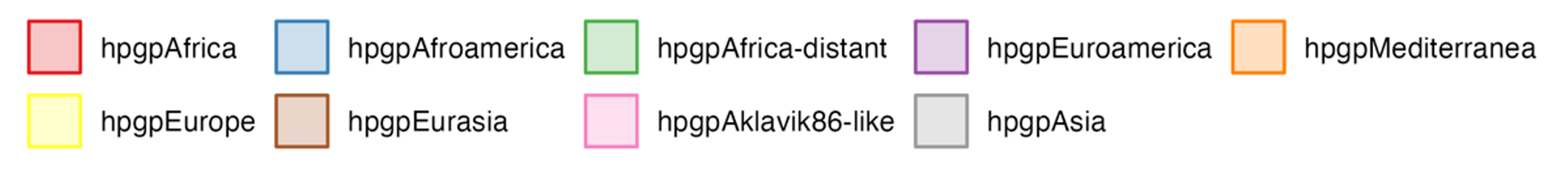


**C**

**B**

Supplemental Figure 2. Piecewise linear correlations of GMM-identified SNP cluster minor allele frequencies with percent European ancestry for Cluster 1 (panel **A**), percent Asian ancestry for Cluster 2 (panel **B**), and percent African ancestry for Cluster 3 (panel **C**).


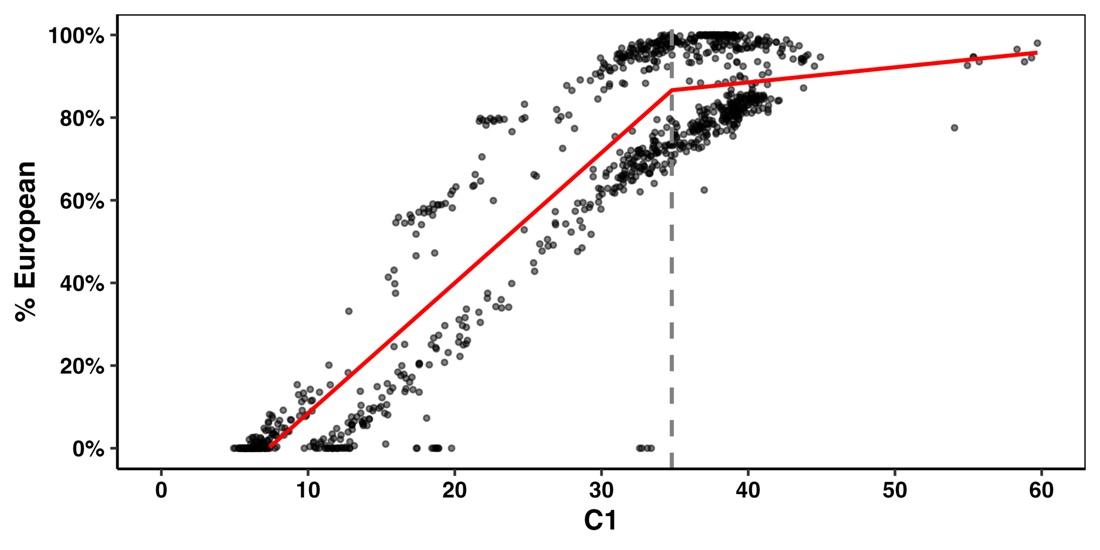


**A**

*r* = 0.92


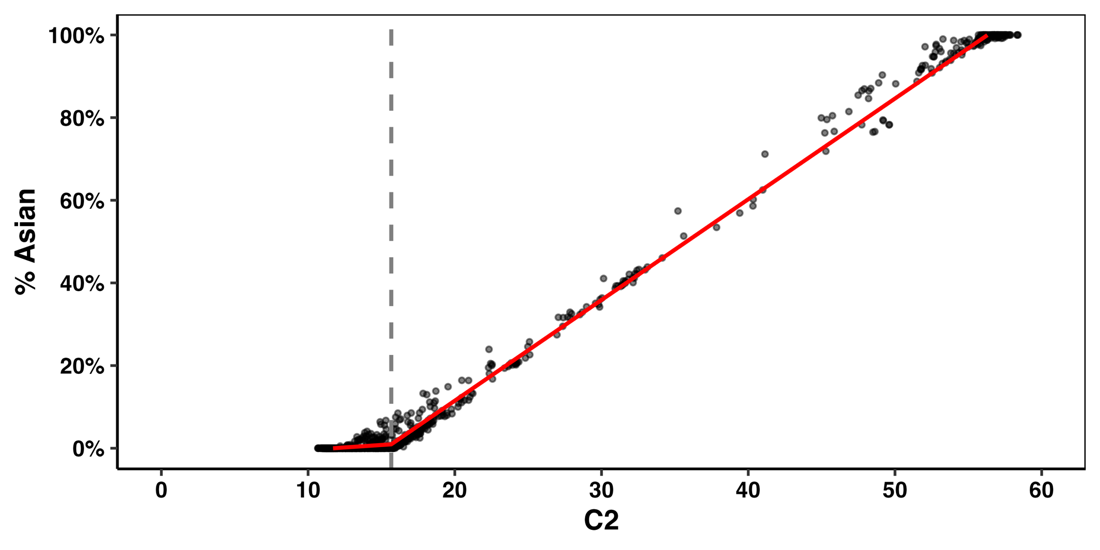


**B**

*r* = 1


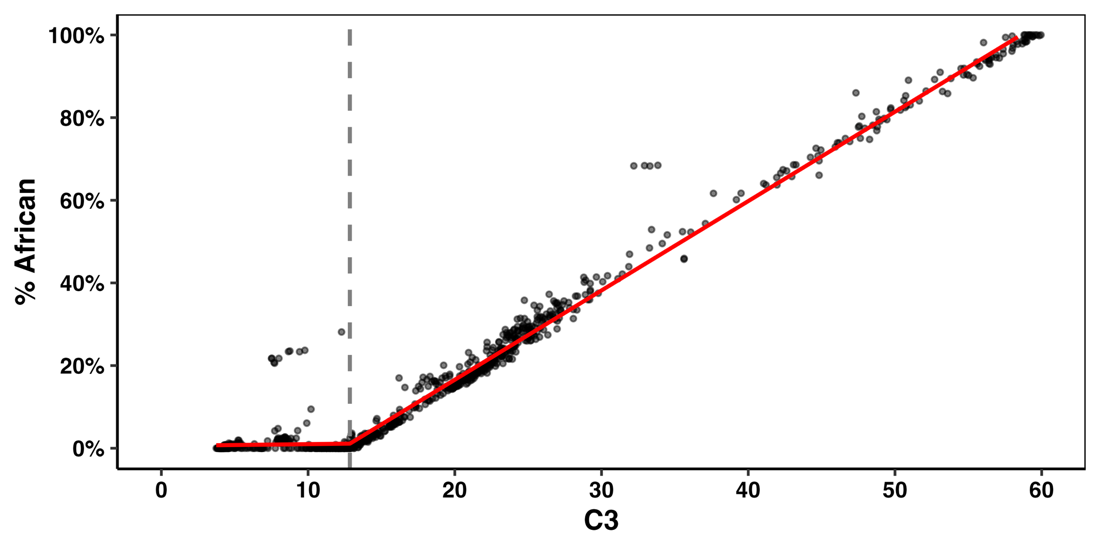


**C**

*r* = 1

Supplemental Figure 3. Classification accuracy of LightGBM models using SNP cluster assignments derived from GMM models with 1 to 10 clusters. For each cluster configuration, model accuracy was averaged across five independent runs. These results were used to identify the optimal number of clusters for downstream predictive modeling.


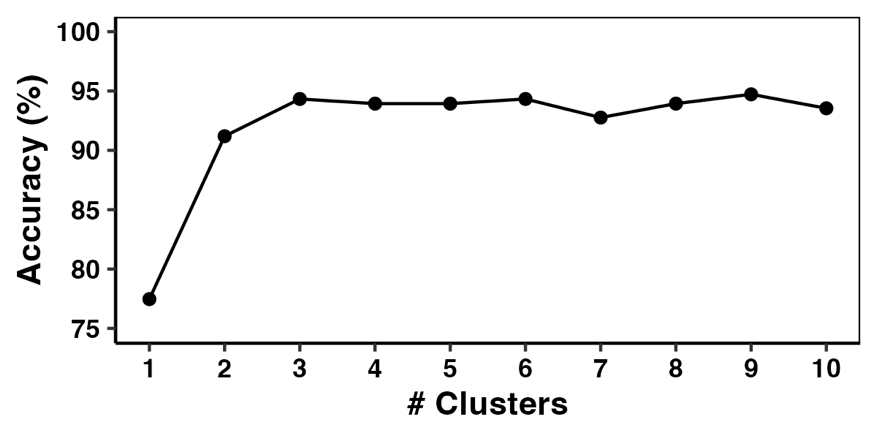


Supplemental Figure 4. Boxplot depicting the predicted probabilities for each test sample from the best-performing LightGBM run. Classification was based on the highest probability among the nine reference population classes. Ten of 255 test samples were assigned to incorrect classes, and their corresponding predicted probabilities are indicated.


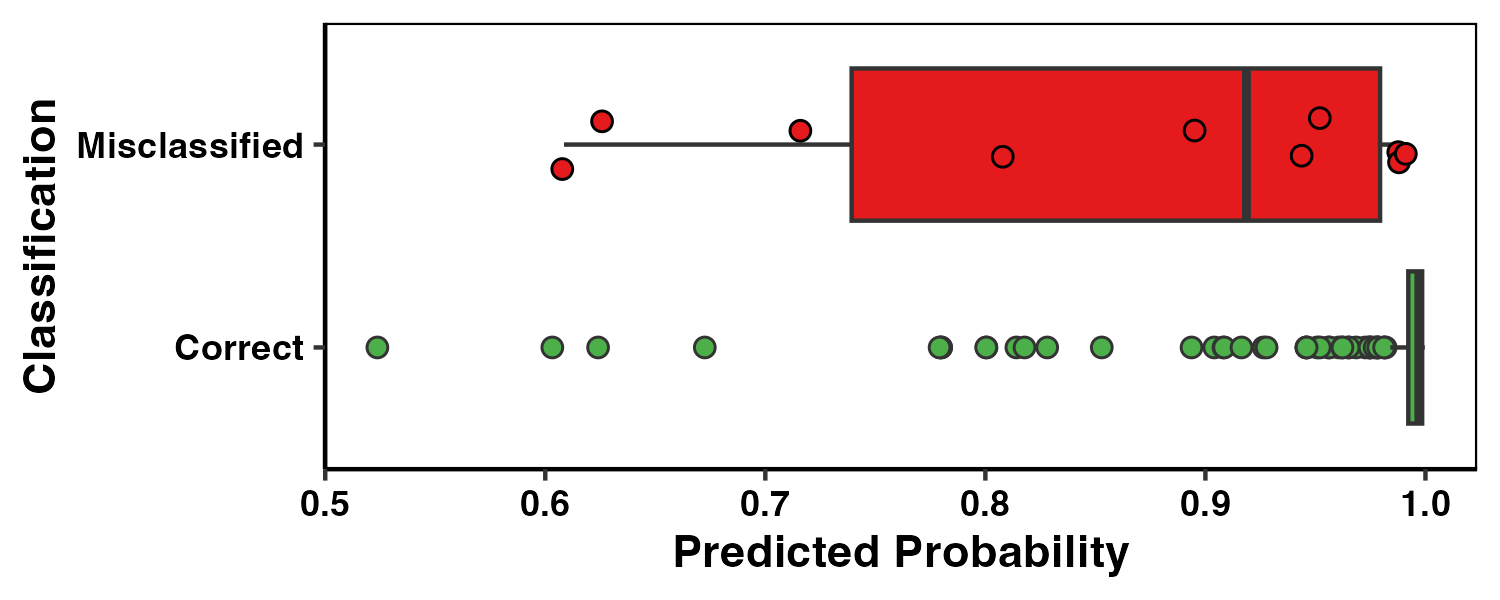


Supplemental Figure 5. Sankey plot for cluster membership flow across methods


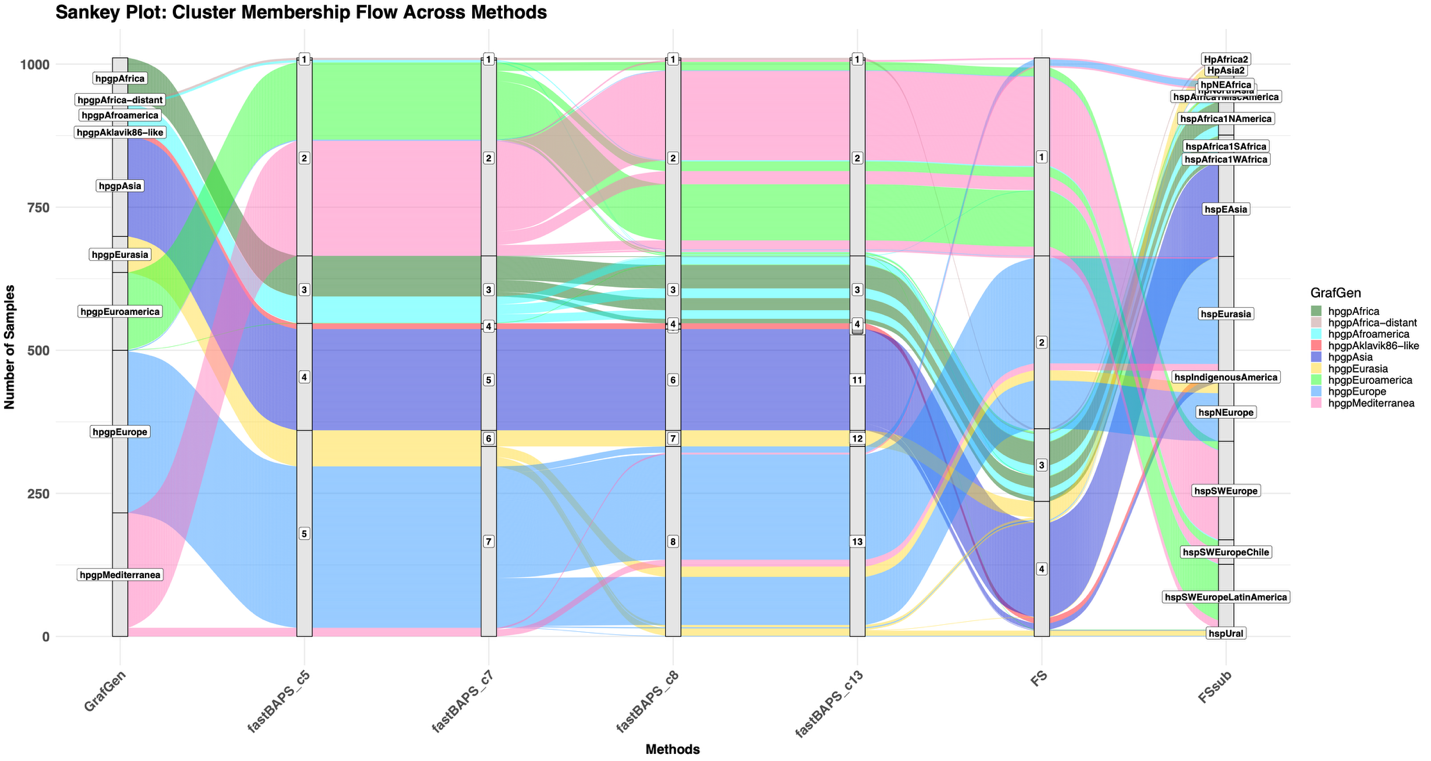


Supplemental Figure 6. Genetic distance-based clustering of 366 *Hp*GP prophage sequences, including 80 complete prophages with preserved synteny, 16 complete prophages with large insertions or deletions, and 270 incomplete prophages


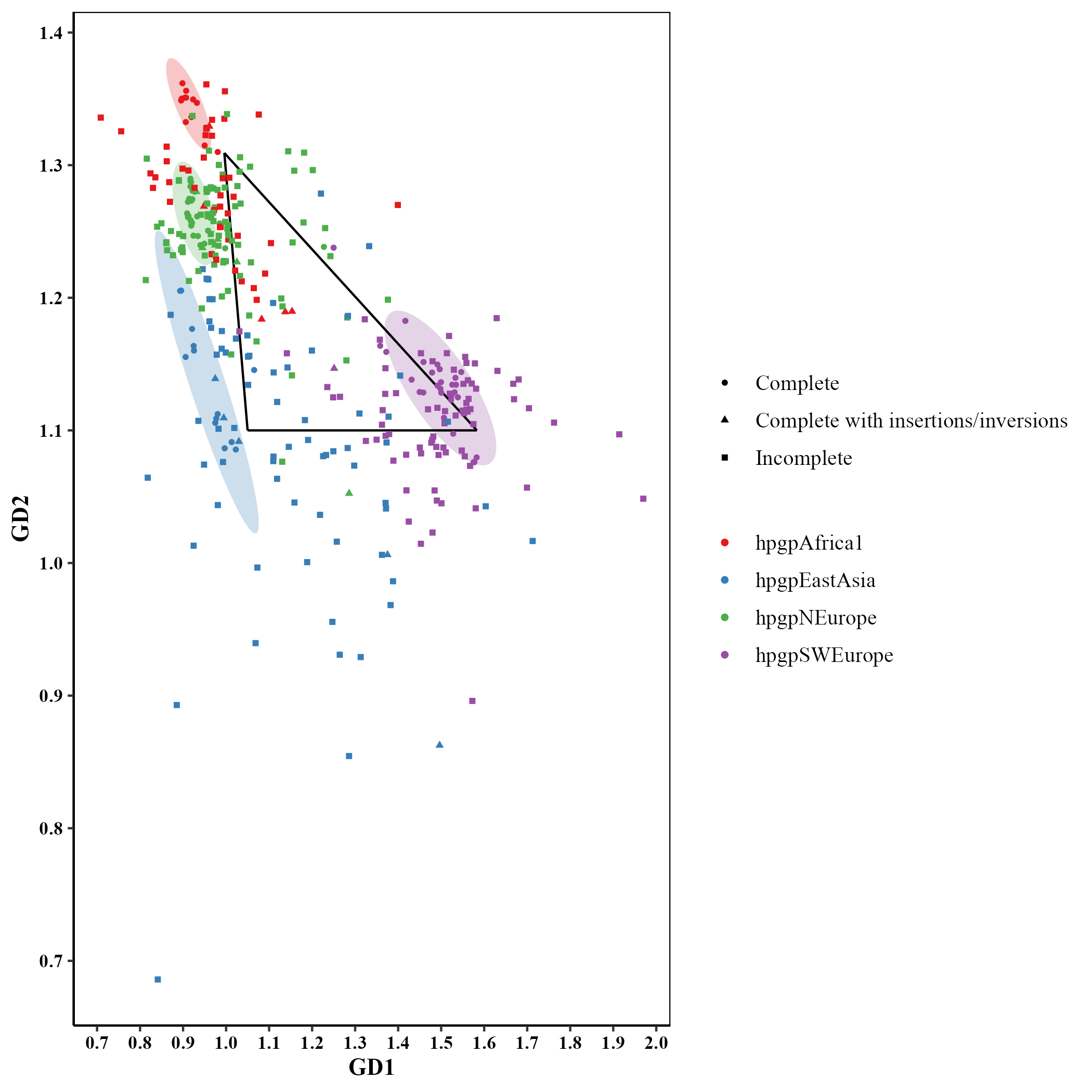


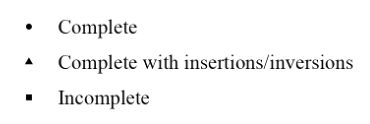

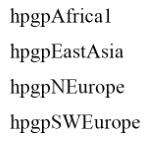

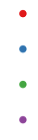
Supplemental Figure 7. Dot plot for the genome comparison between representative ubiquitous (hpgpEurope, hpgpAsia and hpgpAfrica) and all hardy (hpgpAklavik86-like cluster in GrafGen) strains vs. 26695


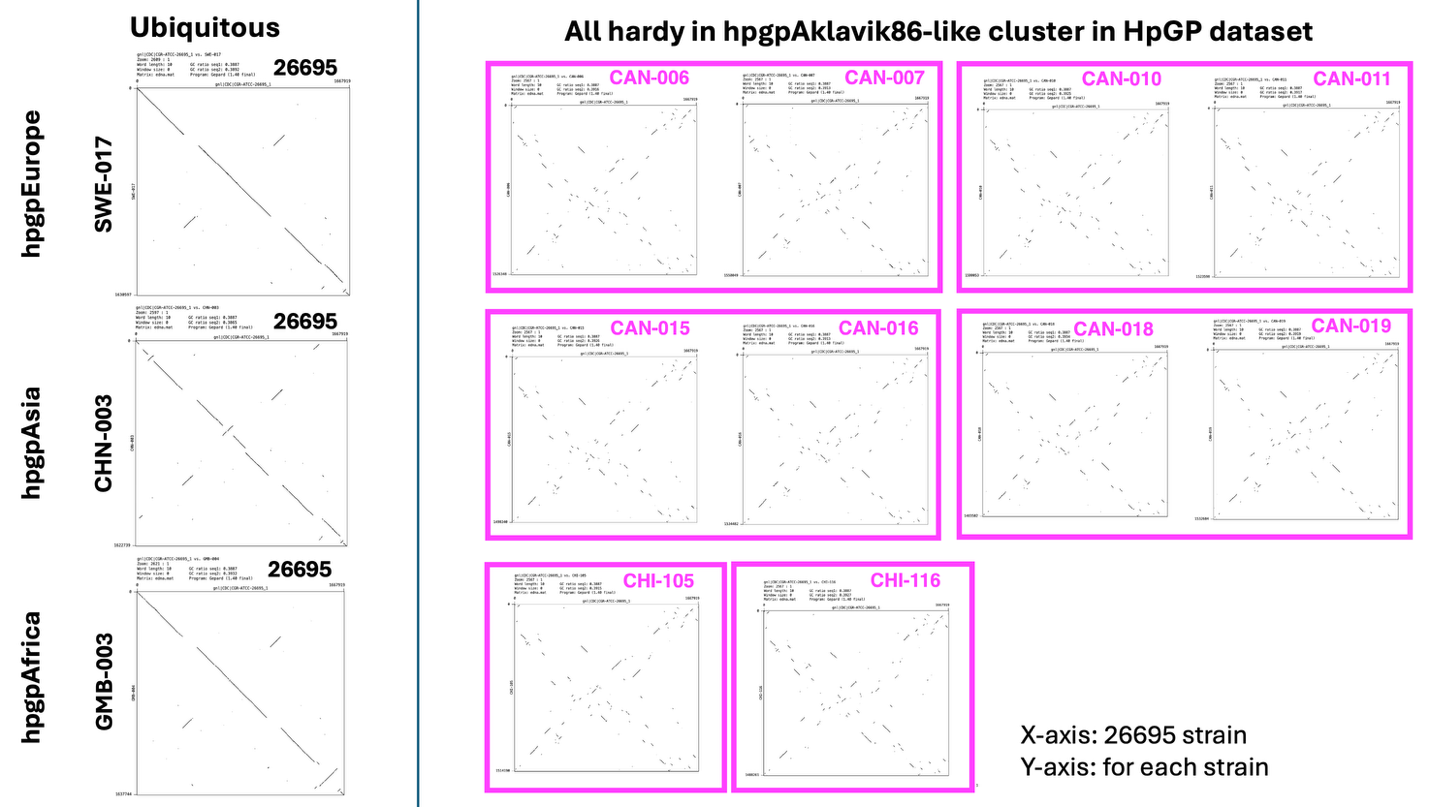


Dot plot was created using Gepard program (v2.1). <https://github.com/univieCUBE/gepard>.

Krumsiek J, Arnold R, Rattei T.  Gepard: a rapid and sensitive tool for creating dot plots on genome scale. Bioinformatics, *23*, 2007, 1026–1028
